# Neonatal Immune Responses to Respiratory Viruses

**DOI:** 10.3389/fimmu.2022.863149

**Published:** 2022-04-14

**Authors:** Taylor Eddens, Olivia B. Parks, John V. Williams

**Affiliations:** ^1^Pediatric Scientist Development Program, University of Pittsburgh Medical Center (UPMC) Children’s Hospital of Pittsburgh, Pittsburgh, PA, United States; ^2^Division of Allergy/Immunology, University of Pittsburgh Medical Center (UPMC) Children’s Hospital of Pittsburgh, Pittsburgh, PA, United States; ^3^Medical Scientist Training Program, University of Pittsburgh, Pittsburgh, PA, United States; ^4^Division of Pediatric Infectious Diseases, University of Pittsburgh Medical Center (UPMC) Children’s Hospital of Pittsburgh, Pittsburgh, PA, United States

**Keywords:** neonate, lung, respiratory virus, RSV, rhinovirus, human metapneumovirus

## Abstract

Respiratory tract infections are a leading cause of morbidity and mortality in newborns, infants, and young children. These early life infections present a formidable immunologic challenge with a number of possibly conflicting goals: simultaneously eliminate the acute pathogen, preserve the primary gas-exchange function of the lung parenchyma in a developing lung, and limit long-term sequelae of both the infection and the inflammatory response. The latter has been most well studied in the context of childhood asthma, where multiple epidemiologic studies have linked early life viral infection with subsequent bronchospasm. This review will focus on the clinical relevance of respiratory syncytial virus (RSV), human metapneumovirus (HMPV), and rhinovirus (RV) and examine the protective and pathogenic host responses within the neonate.

## Respiratory Viral Infections in the Neonatal Population

Global studies estimate that over 100 million lower respiratory tract infections occur annually in children under the age of 5, accounting for 700,000-900,000 deaths per year in this age group (1). The estimated RSV and HMPV lower respiratory tract infection burden in this age group is approximately 33.1 million and 14.2 million cases per year, respectively (2, 3). For context, estimates of influenza lower respiratory tract infection in children under the age of 5 is 10.1 million cases per year (4). RV lower respiratory tract infection burden has not been quantified on a global scale; however, at least in hospitalized children, RV has been shown to be a common cause of bronchiolitis (5–7).

RSV is a negative-sense single-stranded RNA virus in the *Pneumoviridae* family (8). RSV cases typically peak in the winter months in temperate climates (9). RSV is a common pathogen in young children, with one study estimating the infection rate as 68.8/100 children under the age of 1 year with near ubiquitous exposure by 2 years (10). RSV infection typically presents with upper respiratory symptoms, with progression to lower respiratory symptoms in approximately 40% of cases (11, 12). While progression to bronchiolitis requiring hospitalization is relatively rare (1-2% of all RSV cases), RSV accounts for nearly 70% of all hospitalizations for bronchiolitis in the United States (13, 14). Global estimates of RSV disease are staggering, with 3.2 million hospital admissions and 59,600 in-hospital deaths in 2015; 45% of hospitalizations (~1.4 million) and deaths (~27,300) were in infants under the age of 6 months (2). Premature infants are at especially high risk, with one meta-analysis indicating 3-fold higher risk of hospitalization in premature compared to term infants (15).

HMPV is a negative-sense single-stranded RNA virus first isolated from children with lower respiratory tract infection by Dutch investigators in 2001 (16). HMPV is also a member of the *Pneumoviridae* family (16–18). HMPV largely occurs in the winter and early spring months in the United States, typically 1-2 months after the peak of RSV season (19–22). Most children are infected with HMPV within the first years of life, although re-infection can occur frequently with either heterologous or homologous strains of HMPV (22, 23). HMPV infection most commonly presents in children with fever, rhinorrhea, and cough, while wheezing on presentation has been reported in approximately 50% of pediatric cases (22, 24, 25). One prospective multicenter surveillance study demonstrated that HMPV causes 7% of sick clinic and emergency department visits for children <5 years of age (26). In 2018, HMPV infections globally in children under the age of 5 years contributed to approximately 500,000 hospital admissions and 11,300 deaths (3). Infants had a disproportionately high risk of hospitalization and death—a trend that was magnified in low-income or low-middle-income countries (3). Infants with history of prematurity also have increased susceptibility to hospitalization and severe HMPV disease (26–29).

Human rhinoviruses are positive-sense single-stranded RNA virus in the family *Picornaviridae* and genus *Enterovirus* (30–32). Rhinovirus (RV) infection most commonly occurs in the fall with a smaller peak in the spring (33, 34). The mean age for first symptomatic RV infection is approximately 6 months, with re-infection *via* heterologous strains occurring frequently (35, 36). RV infection has a range of presentations: asymptomatic (occurring frequently in young children), upper respiratory tract infection (known colloquially as the ‘common cold’), and lower respiratory tract infection (37–42). However, RV is a frequent cause of lower respiratory tract infection in infants and toddlers, causing common presentations of bronchiolitis, pneumonia, and wheezing episodes (43–50). Hospitalization among children with RV was disproportionately seen in infants <6 months, with RV infection accounting for ~5 out of every 1,000 hospitalizations in this age range (51). Like RSV and HMPV, RV infection can cause severe disease in premature infants; in fact, nosocomial outbreaks of RV have been described in neonatal intensive care units (52–54).

Collectively, RSV, HMPV, and RV represent three leading causes of respiratory morbidity and mortality in young children, highlighting the need to understand host-pathogen interactions in the lung that put these patients at higher risk of poor outcomes. The unique immunologic functions of the neonatal lung in homeostasis shape the responses to these three pathogens.

## Lung Development and Prenatal Immunity

Structurally, the lungs *in utero* undergo three major developmental stages characterized by histologic appearance: pseudoglandular, canalicular, and terminal saccular stages (55). When the lungs spring open with the newborn’s first cry, the primary functions of ventilation and oxygenation begin as residual amniotic fluid is absorbed. However, for the next 4 weeks in mice and the next 1-2 years in humans, the last stage of development, alveolarization, occurs. This process is marked by increased branching and budding of alveoli, exponentially increasing the surface area of cells capable of performing gas-exchange (55).

As infectious threats to the newborn lung can be present from that first breath, it is unsurprising that development of the immune system within the lung occurs early in embryonic development (56). Immune cells, particularly embryonic macrophages and dendritic cells, can be found in the lung as early as embryonic day(E) 9.5 in mice and E35 in humans (57, 58). NK cells are present early in fetal development in the murine liver and spleen at E14.5 and 15.5, respectively (59). Shortly after, γδ T cells develop in the embryonic thymus and are important sources of prenatal IL-17 (60, 61). Proteins like mucins (e.g. Muc5b) and surfactants (e.g. SP-A) are released from the prenatal lung into the amniotic fluid and both substances have immunomodulatory properties (62–66).

## Overview of Lung Innate Immunity in the Neonate

**Figure 1:**
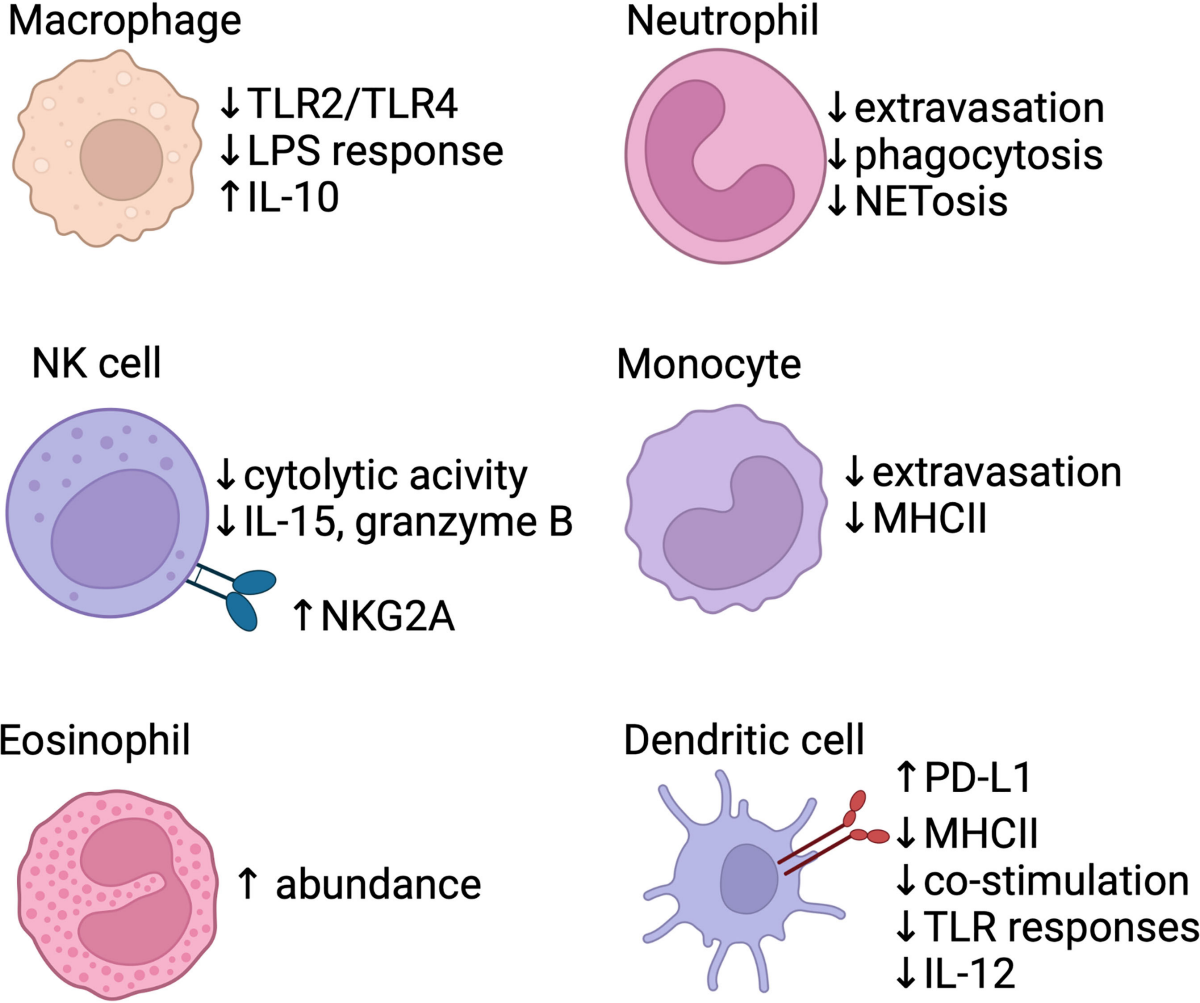
Baseline differences in the neonatal innate immune system. Several areas of the innate response are blunted in neonates, including macrophage response to TLR signals, neutrophil/monocyte extravasation, and poor function of antigen-presenting cells (monocytes/dendritic cells). Anti-inflammatory signals are also produced by the innate system, with IL-10 production *via* macrophages. Dendritic cells express PD-L1, a co-inhibitory receptor, shortly after birth and produce less pro-inflammatory IL-12. NK cells show reduced effector function and increased expression of inhibitory receptors (e.g. NKG2A). Eosinophils accumulate in the lung within the first two weeks of life in mice. Created with biorender.com.

Shortly after birth, the composition of the innate immune system in the lung changes. Single-cell RNA sequencing of post-natal day 1 mice demonstrate the presence of 5 unique subsets of macrophages/monocytes and 3 subsets of dendritic cells (DCs), as well as mast cells, basophils, and neutrophils (67). Interestingly, the monocytes present in the murine lung prenatally differentiate into long-lived alveolar macrophages within the first week of life in a GM-CSF-dependent fashion (68). Following LPS stimulation, murine neonatal macrophages have increased production of IL-10 (an anti-inflammatory cytokine); IL-1, IL-6 and TNF production were significantly decreased following LPS stimulation, potentially due to decreased TLR2 and TLR4 expression (**Figure 1**) (69). Between postnatal day 3-21 in mice, macrophages in the neonatal lung show increased polarization towards an M2-like macrophage phenotype, which is temporally associated with alveolarization (70). In contrast, phagocytic function and bactericidal capabilities of neonatal macrophages isolated from human cord blood are comparable to that of adult cells (71, 72). Human cord blood derived macrophages stimulated with LPS do not decrease their oxygen consumption, indicating an impaired ability to modulate metabolism and effectively activate macrophages, while adult macrophages downregulate oxidative phosphorylation, thus shifting to Warburg metabolism (73).

Human cord blood derived macrophages and monocytes are also less responsive to IFN-γ compared to adult cells (74). In humans, circulating monocytes in the newborn exhibit decreased MHC class II expression, which leads to impaired antigen presenting capacity in tissues (75). Cord blood monocytes have an intrinsic impairment in extravasation under homeostatic conditions when compared to adult monocytes, although activation of endothelial cells by inflammation can improve neonatal monocyte migration (76).

In the newborn mouse, absolute number and function of DCs are decreased compared to the adult lung (77, 78). Neonatal lung DCs in mice promote tolerance by upregulating PD-L1 expression shortly after birth in a microbiota-dependent fashion (79). Human cord blood derived DCs have decreased expression of MHC class II and co-stimulatory molecules such as CD25, CD83, and CD86 and exhibit blunted responses to LPS (80, 81). Following LPS stimulation, human neonatal conventional DCs (cDCs) have impaired transcription of IL12-p35, a subunit of the Th1-potentiating cytokine IL-12p70, although this expression was restored by exogenous IFN-γ administration (82, 83). Similarly, stimulation of human cord blood DCs with TLR7 and TLR9 agonists demonstrated defective type I interferon responses (84, 85). Interestingly, murine neonatal DCs may have a constrained response to TLR stimulation in part due to IL-10 production by CD5^+^ B cells, demonstrating the complex interplay of neonatal innate and adaptive immunity (86, 87). In a human cohort followed prospectively, IFN-α production by DCs increased to adult levels by 1 year of age, IL-10 production declined to adult levels by 2 years, while IL12-p70 secretion remained decreased at 2 years (88). While similarities in decreased co-stimulatory molecule expression and blunted inflammatory responses in DCs have been characterized, other differences between murine and human DCs responses to specific stimulations have been reviewed elsewhere (89). More recently, fate-mapping and sequencing based techniques in mice have demonstrated that cDC2s derived early in life have differing responses to pathogens compared to the adult cDC2s due to an altered cytokine milieu in the lung, rather than inherent pre-programmed differences (90).

Although absolute numbers of neutrophils are comparable to that of older children and adults, neonatal neutrophils exhibit poor chemotaxis, endothelial adhesion, and phagocytic function (91–93). Human neonatal neutrophils neither produce neutrophil extracellular traps (NETs) nor respond to Fas-mediated apoptosis as effectively as adult neutrophils (94–97). One study also identified a unique group of myeloid suppressor cells with a neutrophilic/granulocytic phenotype (Gr-MDSC) in human cord blood; this cell population was found to suppress NK cell activation and T cell polarization to effector subsets (98).

NK cells in the human lung exhibit a selective tolerogenic phenotype where they retain their ability to generate strong antibody-dependent cell-mediated cytotoxicity (ADCC) responses similarly to adult NK cells, but are unable to mount a response against cells displaying non-self peptide or lacking MHC class I receptor (99). Analysis of human cord blood revealed that NK cells have increased expression of inhibitory receptors (e.g. NKG2A/CD94) and decreased expression of activation receptors (e.g. leukocyte immunoglobulin-like receptor (LIR)-1), leading to impaired recognition of MHC-I negative cells (100–102). Human cord blood derived NK cells also had decreased toll-like receptor 3 (TLR3) expression and failed to mount a response to polyinosinic-polycytidylic acid (poly(I:C)), a synthetic viral double-stranded RNA viral pathogen-associated molecular pattern, but paradoxically had increased IFN-γ release with TLR8 stimulation (103, 104). NK cells isolated from cord blood also had a 3-fold lower capacity for cytolytic activity and decreased degranulation and cytokine production (e.g. IL-15, IL-2, granzyme B, and IFN-γ) compared to adult NK cells (100, 105–107). Maturation of neonatal murine NK cells into adult-like NK cells was constrained by TGF-β signaling (108).

While comparing murine-derived data from lung tissue with peripherally acquired human cells presents a challenge, a pattern of anti-inflammatory and tolerogenic responses from neonatal innate immune cells emerges. Stimuli that would normally initiate a robust inflammatory response in adult models, such as LPS or other pathogen-associated molecular patterns, have blunted effects on neonatal dendritic cells and macrophages (69, 73, 82–86). Further, innate immune cells in the neonate contribute to the local anti-inflammatory milieu by production of IL-10 with suppression of ‘typical’ inflammatory cytokines (e.g. IL-12), upregulation of co-inhibitory receptor (e.g. PD-L1) expression, and reduced co-stimulatory receptor expression (69, 79–83, 88).. Collectively, these alterations not only stifle an inflammatory innate response in the neonatal lung, but also effect downstream adaptive immunity as well.

## Overview of Lung Adaptive Immunity in the Neonate

Although once thought to be in a relative state of immunosuppression due to poor adaptive immunity, recent studies clearly demonstrate the presence of a more nuanced adaptive immune response in the neonate (109–112). Single cell RNA sequencing demonstrated T cell and B cell numbers increase in the murine neonatal lung on the first day of life (67, 113). This early arrival in the lungs is not necessarily in response to a stimulus, but reflective of the fact that neonatal naïve T cells extravasate into end-organ sites to a higher degree than adult naïve T cells (114–116). Following trafficking to the lung, the adaptive immune system of the neonate has strikingly different properties than adult counterparts (117, 118). This concept has been described as a ‘layered immunity,’ as neonatal B and T cells have functional differences that phenotypically distinguish these cells (119–123)..

**Figure 2:**
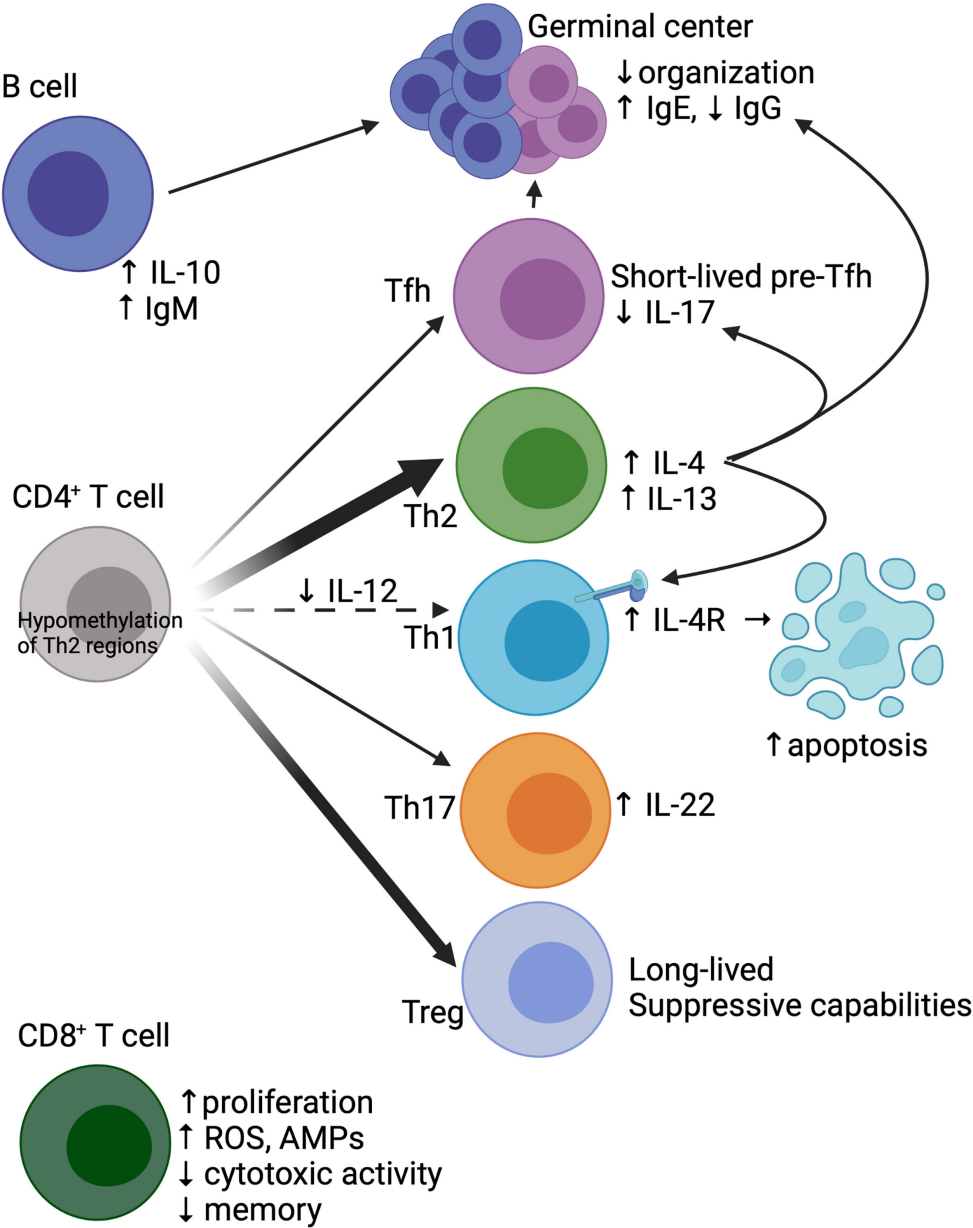
Baseline differences in the neonatal adaptive immune system. The neonatal CD4^+^ T cell compartment is skewed towards Th2 (due to hypomethylation of critical Th2 regulatory regions) and Treg development. There is less differentiation towards Th1 due to reduced IL-12 in the milieu, coupled with increased Th1 apoptosis due to IL-4 signaling. Tfh cells, while stimulated by IL-4 to differentiate, have arrested development, with generation of short-lived pre-Tfh cells. IL-4 signaling on Tfh cells also limits IL-17 production and skews the humoral response towards IgE production. Both neonatal Tfh and B cells have poor migration to germinal centers, which structurally demonstrate poor organization. B cells also have increased production of IL-10 and spontaneous secretion of IgM. CD8^+^ T cells show radically different properties in neonates compared to adults, with increased proliferation, generation of reactive oxygen species (ROS) and antimicrobial peptides (AMPs), and reduced cytotoxicity and memory formation. Created with biorender.com.

While CD8^+^ T cells are present in the murine newborn lung at a relatively low abundance, exposure to pathogens can lead to rapid induction of CD8^+^ T cells (67, 113, 124, 125). Neonatal CD8^+^ T cells demonstrate greater proliferative capabilities with rapid terminal differentiation at the expense of memory formation (**Figure 2**) (123, 126–128). From a functional perspective, neonatal CD8^+^ T cells demonstrate decreased cytotoxicity but increased innate-like characteristics, such as antimicrobial peptide production and reactive oxygen species formation (118, 129). Furthermore, antigen-naïve murine CD8^+^ T cells in the neonate can secrete IFN-γ in response to cytokine stimulation alone, suggesting less reliance on TCR signaling (118, 123). IL-12 in particular appears to be a key cytokine in changing the epigenetic landscape of human neonatal CD8^+^ T cells, shifting functionality towards a more ‘adult’ phenotype (130, 131).

Similar to the findings of neonatal CD8^+^ T cells, neonatal CD4^+^ T cells have greater proliferative capabilities (117, 132), a more restrictive T cell receptor (TCR) repertoire with bias towards self-reactive TCRs (133–135), and are more likely to become terminally differentiated rather than form a memory population (136). However, neonatal CD4^+^ T cells have significant heterogeneity in differentiation states (e.g. subsets) and effector function. Compared to adult cells, murine CD4^+^ T cells found after birth skew towards Th2 and away from Th1 cells (**Figure 2**) (137–139). Neonatal CD4^+^ T cells produce an abundance of IL-4 and IL-13 after activation, in part due to hypomethylation of a key murine Th2 cytokine regulatory region (CNS-1) and an open IL-13 locus resistant to transcriptional repression in human cells (140–142). This epigenetic pattern in T cells in mice changes rapidly post-birth, reaching adult-like epigenetic patterns within 6 days of life and thus contributing to this notion of a “critical window” of Th2 bias (143). In murine Th1 cells, lack of IL-12 in the milieu leads to upregulation of IL-13Rα1, allowing for IL-4 signaling through the IL-4 receptor which results in induction of apoptosis, furthering the imbalance of Th2/Th1 cells (144–146).

In addition to favoring the Th2 subset, naïve T cells from the neonate preferentially differentiate into regulatory T cells (Tregs) (117, 132). Using a fate-mapping Foxp3 mouse, Tregs generated early in life in the lung demonstrated suppressive activity that was maintained into adulthood (147). Similarly, in humans, Tregs directed against maternal alloantigens could be detected in children 7-17 years old, suggesting a long-lived functionality of neonatal Tregs (148). To a lesser extent than Th2/Treg skewing, one murine fate-mapping model showed increased neonatal T cell differentiation towards Th17 cells (149). Human cord blood derived T cells cultured in Th17-polarizing conditions produced higher levels of IL-22, an anti-inflammatory cytokine designed at maintaining epithelial barriers, when compared to adult T cells (150).

T follicular helper (Tfh) cells, a Bcl6-dependent CD4^+^ T cell subset that specializes in facilitating germinal center reactions and B cell development, also show altered function in neonates (151). In studies of neonatal immunization, induction and migration of Tfh cells were impaired compared to adult mice (152, 153). Further, impaired Tfh cell function in neonates led to reduced germinal center quantity, less differentiation of B cells towards plasma cells, and reduced antigen-specific IgG production when compared to adult mice (152). While neonatal Tfh cells demonstrate some degree of intrinsic dysfunction, the extrinsic milieu in the neonate also plays a factor; for instance, IL-4 seems to promote Tfh cell development and localization to germinal centers, while simultaneously constraining IL-17 production by Tfh cells and skewing the humoral response towards IgE production (152, 153). Using microarray, a more recent study demonstrated that neonatal Tfh cells can initiate the transcriptional program associated with Tfh development, but preferentially differentiate into short-lived pre-Tfh cells more so than adult counterparts (154). Further, these neonatal Tfh cells also expressed a signature more classically associated with Th2 cells, such as increased expression of IL-13 and transcription factors associated with Th2 development (154).

While the diminished function of Tfh cells in neonates contributes to the changes in the humoral response, there are also key differences between neonatal and adult B cells. The murine and human neonatal B cell compartment is largely composed of a first layer of B-1 B cells arising from the embryonic yolk sac and fetal liver, compared to mature B-2 cells in the adult (120). Murine B-1 cells, unlike B-2 cells, do not require IL-7 or B cell activating factor (BAFF) for development and are more responsive to TSLP (155, 156). Neonatal B cells in mice also have low levels of activation induced cytidine deaminase (AID) leading to lower affinity antibody responses (157). Human cord blood derived B-1 cells have a more restricted immunoglobulin repertoire, spontaneously secrete IgM, and exhibit limited somatic hypermutation (158). Passive humoral immunity, though, is provided *via* transplacental transfer of high-titer maternal antibodies, which occurs in humans during the third trimester of pregnancy (159–161).

Taken together, these studies not only indicate that the neonatal lung has a coordinated first layer of adaptive immunity at baseline, but are also identifying the mechanistic differences between neonatal and adult adaptive immunity. The evolutionary reasons for these altered responses remains an active question. Considering the altered CD4^+^ T cell response as a prototypical example, the Th2-predominance may be critical for lung development, as evidenced by the temporal association of polarization of M2-like macrophages and recruitment of eosinophils with alveolarization and primary septation, respectively (70, 162). Additionally, there is a theological argument to be made for limiting a potentially inflammatory and cytotoxic Th1 response in area of active development. While sustaining a milieu favoring Th2 vs. Th1 cells in the neonatal lung under physiologic conditions presumably confers some evolutionary advantage to the host, understanding this baseline difference—along with the others discussed in these last two sections—informs how the neonatal immune system responds in the face of a viral infection.

## Neonatal Immune Responses to Respiratory Viral Infection in Preclinical Models

Several neonatal animal models exist to study the host-pathogen interactions of respiratory viruses, with the most extensive body of literature surrounding neonatal RSV infection.

**Figure 3:**
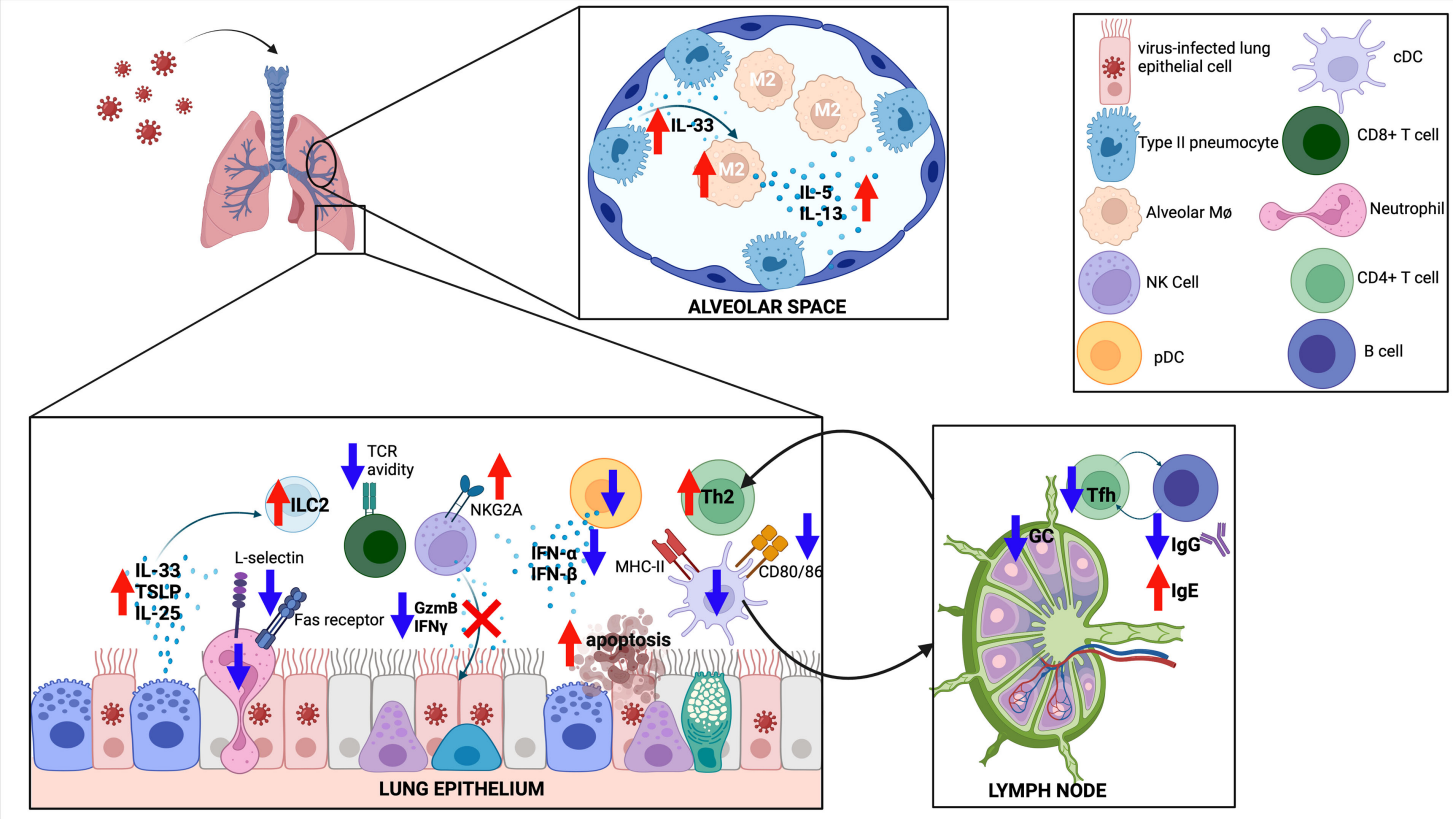
The immune response to respiratory viruses in neonates. The neonatal response favors a type II response, with increased IL-33, IL-25 and TSLP released from the epithelium, increased type 2 innate lymphoid cells (ILC2), increased type 2 helper T cells (Th2), and differentiation of M2-like alveolar macrophages (M2). CD8 T cells have reduced T cell receptor (TCR) avidity, while both CD8 T cells and natural killer (NK) cells show reduced effector functions. Germinal center (GC) reactions are diminished in neonates, with less T follicular helper (Tfh) cell differentiation and less IgG production from GC B cells. Created with biorender.com.

RSV infection of newborn mice resulted in prolonged viral replication when compared to adult mice, although neonates are capable of controlling and clearing infection (163, 164). Neonatal epithelial cells release IL-33 and TSLP in greater magnitude following RSV infection compared to adult mice (**Figure 3**) (165, 166). Early mediators of antiviral response such as recruitment of plasmacytoid DCs (pDCs) and production of IFN-α and IFN-β were deficient in neonatal mice infected with RSV (167). cDC1s were adequately recruited to the lung, but failed to respond to type I IFNs and upregulate co-stimulatory molecules (e.g. CD80/CD86) required for proficient CD8^+^ T cell response following RSV infection in mice (168). cDCs from human cord blood infected with RSV similarly had poor co-stimulatory molecule expression (168). Antigen-specific CD8^+^ T cells were induced in neonatal mice following RSV infection but were less likely to produce IFN-γ (169). This lack of IFN-γ production leads to a delayed and blunted classically activated macrophage response in neonatal mice (163). TLR4 or TLR9 agonist treatment increased co-stimulatory molecule expression on murine cDCs and restored CD8^+^ T cell responses to adult levels (170). Interestingly, the CD8^+^ T cell repertoire following RSV infection is significantly different in neonatal mice with variations in epitope-specific Vβ repertoire usage and lower functional avidity, a finding that resolves within 2 weeks of life, highlighting a critical window for altered CD8^+^ T cell responses (164).

Neonatal RSV infection in mice was associated with recruitment of CD4^+^ T cells with a Th2 bias (171). Compared to adult mice, the CD4^+^ T cell compartment of neonatal mice showed increased proliferative capacity, expression of IL-4Rα, and differentiation into Th2 cells following RSV infection (172). Using a lamb model, neonatal RSV infection further increased Th2 cytokine production in the setting of an elevated baseline Th2 milieu (173). This skewing towards type 2 inflammation (e.g. increased ILC2s and Th2s) was due to rapid and increased production of IL-33 in neonatal mice (165, 174). Consistent with a Th1/Th2 imbalance, neonatal mice had fewer Th1 CD4^+^ cells in the bronchoalveolar lavage fluid when compared to adult mice (171).

RSV infection of 7-day-old mice resulted in long-standing pathophysiologic changes such as reduced lung function, chronic inflammation, and airway remodeling; synergistic changes were noted in mice sensitized to OVA after prior neonatal RSV infection (175). Re-infection 4-6 weeks after initial neonatal infection led to an exaggerated Th2 response and increased airway hyperresponsiveness when compared to weanling-aged or adult-aged mice infected twice with RSV (172, 176). Mechanistically, neonatal mice with repeated exposure to RSV demonstrated a break in tolerance to an antigen (OVA) present in maternal breastmilk, increasing susceptibility to allergic airway disease (177). It should also be noted that RSV strain virulence could also play a role in mediating pathology, as a chimeric RSV A2 strain carrying the F protein from a clinical isolate induced a more robust Th2 response and subsequent lung pathology (178).

Murine Th2 responses following neonatal RSV infection contribute to pathology. CD4^+^ T cell-specific deletion of IL-4Rα led to reduced Th2 responses and reduced airway hyperresponsiveness following secondary exposure (172). Similarly, administration of an IL-4Rα antisense oligonucleotide during the initial neonatal infection mitigated Th2-mediated pathology with subsequent RSV exposure (179). Interestingly, administration of recombinant IFN-α during primary infection led to reduced pathology with re-infection, in part by limiting increased IL-4Rα on Th2 cells (167). Amelioration of altered airway responses after secondary infection could also be achieved with administration of an IL-13Rα2 fusion protein, blockade of IgE, blockade of IL-33 signaling, or inhibition of STAT6 during the primary infection (165, 176, 180, 181).

The lack of a strong Th1 response in neonates also may contribute to pathology. RSV-infected human cord blood DCs produce increased TGF-β, an anti-inflammatory cytokine that in this context limits IL-12p70 production (and subsequent Th1 differentiation), compared to RSV-infected DCs derived from adults (182). Recombinant IFN-γ therapy diminished the number of GATA3^+^ CD4^+^ T cells (Th2) and increased antigen-specific CD8^+^ T cell recruitment to BAL fluid (171). Recombinant IFN-γ treatment in mice activates classically activated and alveolar macrophages and leads to improved RSV clearance (163, 183). Likewise, neonatal infection with a recombinant RSV strain expressing IFN-γ led to improved viral clearance with primary infection and reduced pathology upon secondary infection (184). Collectively, these studies suggest that diminishing an exuberant Th2 response or enhancement of a deficient Th1 response can limit RSV-induced pathology.

Type 17 immunity has been implicated in protection and pathology in neonatal RSV infection. To recapitulate the clinical phenomenon of infants with CX3CR1 gene variants having worse outcomes with RSV infection, Das et al. evaluated neonatal RSV infection in mice lacking this chemokine receptor (185, 186). *Cx3cr1* knockout mice also had worsened pathology dependent on increased IL-17 from γδ T cells (185). Administration of IL-22, a cytokine produced by Th17 cells, Th22 cells, and others to neonatal mice infected with RSV led to faster reduction in RSV burden by limiting RSV-mediated subversion of autophagy (187). RSV infection of a subgroup neonatal B cells named B regulatory cells (nBregs) derived from human cord blood occurs through interaction with the chemokine receptor CX3CR1; infection of nBregs led to secretion of IL-10, further blunting the Th1 response (188).

From a humoral perspective, neonatal mice produced RSV-specific IgE after infection, consistent with a Th2 bias (181). However, neonatal mice had a less robust RSV-specific neutralizing IgG response compared to adult mice (189). Interestingly, depletion of CD4 and CD8 T^+^ cells, NK cells, or IFN-γ blockade led to enhanced RSV-specific antibody production in the neonates (189). The poor antibody response in neonates was the result of poor germinal center activity and deficient differentiation of CD4^+^ T cells to the follicular helper (Tfh) subset (190). Mice lacking Tfh cells had increased pathology with re-exposure to RSV, while blockade of IL-2 led to increased Tfh number, reduced RSV-driven pathology, and increased RSV-specific IgG production (190). Interestingly, administration of recombinant IFN-α during neonatal RSV infection led to increased B cell trafficking to the lung, increased B-cell activating factor (BAFF) and a proliferation-inducing ligand (APRIL) expression, and increased RSV-specific IgA (191).

Another member of the *Pneumoviridae* family, pneumonia virus of mice (PVM), has been utilized as a murine model of early life lower respiratory tract infection (192, 193). Unlike RSV or HMPV, however, PVM is a natural murine pathogen; this allows for the investigation of infection dynamics and the resultant immune response in context of a natural host-pathogen dyad (192). Although kinetics of PVM infection were similar between neonatal and adult mice, neonatal mice had markedly reduced pro-inflammatory mediator production and leukocyte recruitment (194). In a model of severe bronchiolitis using IRF7-deficienct mice, neonatal infection with PVM led to release of IL-33 and HMGB1 (high-mobility group box 1), another nuclear alarmin, both of which contributed to ILC2 proliferation, type 2 inflammation, and airway remodeling (195). Similar to neonatal models of RSV, early life PVM coupled with exposure to an allergen led to Th2-driven phenotype mirroring asthma (196, 197).

The relationship between neonatal infection and type 2 inflammation has also been well characterized in a neonatal RV model. RV infection in mice at DOL7 led to detectable viral RNA for 7 days, accompanied by inflammatory cell infiltration and upregulation of IL-13 in the acute phase (198). However, pathology persisted 4 weeks after initial exposure, as mice with neonatal RV infection had exaggerated airway hyperresponsiveness compared to age-matched uninfected controls *and* RV-infected adult mice (198). Interestingly, these late-phase pathologic effects were mitigated by anti-IL-13 treatment in the immediate post-infectious period (198). Neonatal RV infection resulted in induction of IL-33, IL-25, and TSLP–all of which are epithelial derived cytokines implicated in asthma pathogenesis–and in this model contributed to type 2 innate lymphoid cells (ILC2) expansion (199–201). Neonatal infection with RV followed by a challenge with a heterologous strain of RV lead to increased expansion of this pathologic ILC2 population, perhaps serving as a murine model of the clinical response in children with frequent RV re-exposure (202). Additionally, sensitization to an unrelated antigen (e.g. OVA) followed by antigen challenge led to worsening airway hyperresponsiveness in mice with a history of neonatal RV infection (198). There were strain differences observed as well, as neonatal RV-C infection led to an enhanced type 2 response when compared to RV-A infection, in part due to poor inflammasome activation and decreased IL-1β production in the RV-C infected animals (203). Mitigation of this strong type 2 inflammatory response could be achieved with recombinant IFN-γ treatment, demonstrating a reciprocal relationship between type 1 and type 2 immunity (204).

Limited preclinical studies exist on neonatal HMPV infection, although unpublished data generated in our laboratory suggest neonatal mice are capable of HMPV clearance similar to RSV models. In children less than <3 years of age with a documented HMPV infection, nasal secretions showed a relative increase in proteins associated with Th1 responses but not Th2 responses; this deviation in Th1/Th2 balance was abrogated in patients with a history of prematurity (205). In contrast, a second study evaluating nasal protein levels found infants with HMPV infection had a decrease in IFN-γ/IL-4 ratio (e.g. Th2-skewing) when compared to RSV and influenza (206).

In summary, the preclinical neonatal models of respiratory viral infection demonstrated an amplification of the baseline neonatal immune response: dampened early antiviral pro-inflammatory mediators, reduced co-stimulatory help *via* antigen-presenting cells, poor organization of Tfh/B cell interactions, and reinforced dominance of type 2 immunity. The latter is driven by epithelial-derived cytokines like IL-33, IL-25, and TSLP and contributes to longstanding pathophysiologic changes in some models. Addition of another antigen or allergen synergistically contributed to asthma-like pathology. Collectively, these models demonstrate a skewed, but protective, immune response in neonates compared to adult animals, and help establish a mechanistic link between respiratory viral infection early in life and long-term sequelae like asthma.

## Long-Term Clinical Ramifications of Early Life Respiratory Viral Infection

Several epidemiologic studies have supported the clinically observed phenomenon of development of asthma after an early respiratory viral infection, with a particular focus on RV and RSV (207, 208). Interestingly, first-time wheeze in young children was predominantly associated with RV (44, 209). A prospective study of children showed that wheezing with a RV infection in infancy was associated with a significantly higher risk of wheezing at age 3 and a 10-fold increase in risk of diagnosis of asthma at the age of 6 (210, 211). Similarly, a prospective study of children presenting with bronchiolitis used machine learning clustering to identify risk factors for recurrent wheeze; RV detection was the strongest single predictor (212). A prospective study found RV-C bronchiolitis in infancy, but not RV-A or -B, was associated with recurrent wheeze and IgE-sensitization compared to infants with RSV bronchiolitis (213). Genetic-environment interactions play a role in this process, as RV-related recurrent wheeze (but not RSV) was associated with polymorphisms in the 17q21 locus, a well-established susceptibility locus for development of childhood asthma (214, 215).

Similar to RV, an RSV illness with wheeze early in life also showed a significant 3-fold increase in risk of subsequent asthma development (209, 211). Children with RSV-bronchiolitis that went on to develop asthma were more likely to have an elevated IgE, higher birth weight, or delivery *via* caesarean section (216). At age 18, children with a history of RSV bronchiolitis within the first year of life had an increased risk of asthma, allergy, and sensitization to perennial allergens compared to controls; these differences were magnified when accounting for parental history of asthma (217). Further endotyping of children with RSV bronchiolitis found those with parental asthma, RV co-infection, IgE sensitization, a *Moraxella*-dominant airway microbiome, and high IFN-γ responses had the highest risk of subsequent asthma development, highlighting the myriad factors contributing to this outcome (218). One study of Danish twins found hospitalization with RSV was associated with increased short-term risk of admission for asthma; asthma admission was also associated with severe RSV, demonstrating a bidirectional association, emphasizing a genetic component (219). A second twin study found that RSV hospitalization in early childhood may not directly cause asthma but may indicate a genetic predisposition for subsequent asthma development (220). Large systematic reviews of studies evaluating risk of asthma after RSV infection during infancy demonstrate a higher prevalence of asthma throughout childhood years (221).

Like RSV and RV, HMPV in childhood is associated with development of asthma. Children followed prospectively after a HMPV lower respiratory tract infection were found to have a shorter duration of time between wheezing episodes (both with and without a subsequent viral trigger) when compared to controls without evidence of HMPV infection (222). Furthermore, patients with a history of HMPV bronchiolitis were much more likely to have asthma by age 5 (odds ratio=5.21) compared to patients without HMPV bronchiolitis (223). Additionally, HMPV lower respiratory tract infection in premature infants was associated with abnormal lung function at one year of age (224). In regards to upstream mediators of asthma-like inflammation, HMPV has been shown to induce *in vitro* expression of IL-33 and TSLP in human alveolar epithelial cells (225). Clinically, serum levels of TSLP in children with wheeze during HMPV infection were elevated, further potentiating a link between TSLP production and HMPV infection (226).

While specific pathogens have been evaluated, Bønnelykke et al. recently demonstrated that *any* lower respiratory tract infection (e.g. viral or bacterial) in the first years of life and the frequency of infections were variables associated with increased risk of asthma at age 7 (227). Additionally, while the focus of this review is on infectious triggers of asthma, childhood-onset asthma represents a multifactorial disease (e.g. genetic, exposures, atopy, microbiome etc.) contributing to pathogenesis (228).

## Treatment/Prevention

There is a relative dearth of antiviral agents directed against the respiratory viruses reviewed here. Aerosolized ribavirin has been studied in severely ill children with RSV but has demonstrated minimal efficacy (229). No licensed therapeutic options currently exist for HMPV or RV (18, 30).

Given the lack of effective therapies, the focus has turned to prevention. Vaccination against these respiratory pathogens has been an area of particular emphasis. However, the turbulent experiences of the formalin-inactivated RSV vaccine in the 1960’s delayed progress (230). Infants and toddlers who received the formalin-inactivated RSV vaccine demonstrated worsened outcomes of RSV infection, with 80% requiring hospitalization and two succumbing to infection (231–233). Studies have demonstrated a role for Th2-bias leading to the pathology of this enhanced respiratory disease (ERD); this theory was recently strengthened further after transcriptomic analysis of autopsy specimens from the two fatal cases of ERD in toddlers showed a Th2-signature and low-affinity antibodies causing complement deposition (169, 234–237). Several candidate vaccines, including live-attenuated, inactivated, particle-based, and subunit based, are in preclinical development or clinical trials (233, 238). An alternative approach has focused on maternal immunization with passive immunity conferred to the infant; a nanoparticle protein-based vaccine administered in the third trimester showed a significant reduction in hospitalization rate with RSV in the first 90 days of the infant’s life (239). This would, however, be of less utility for premature infants delivered prior to vaccine administration. HMPV vaccines, including protein-based vaccines, live attenuated viruses, and virus-like particles, have shown promise in animal models (240–247). However, one clinical trial of a live attenuated HMPV vaccine showed only modest induction of a neutralizing antibody response in 30% of participants (248). While RV vaccines have proven difficult given the number of serotypes, several approaches are being explored in preclinical models (30, 249, 250).

A degree of prevention has been achieved against RSV with the use of monoclonal antibodies. Palivizumab, a humanized monoclonal antibody directed against the F protein of RSV, decreased hospitalizations in high-risk infants (e.g., prematurity, congenital heart disease, immunodeficiency) when administered monthly (251–253). However, cost limits the widespread use of palivizumab (251, 254). Several other anti-RSV monoclonal antibodies are in development, including a long-acting monoclonal (nirsevimab) capable of offering protection for 5 months (230, 255). One recent study estimated that a strategic switch from monthly monoclonal injections to either a maternal immunization strategy and/or use of long-acting monoclonals would afford significant cost-savings while providing a similar degree of benefit (256).

Analogous to palivizumab, human monoclonal antibodies against HMPV have been developed; in preclinical models, these monoclonals have been shown to have preventative and therapeutic potential (257, 258). Interestingly, certain HPMV-derived monoclonals show a degree of cross-protection against other *Pneumoviridae* family members, including RSV (259, 260). Development of monoclonal antibodies directed against RV has been studied, but face similar hurdles as RV vaccine development (261).

While the search for a safe and effective vaccine and further monoclonal antibodies continues, the COVID-19 pandemic has also illustrated the efficacy of non-pharmaceutical public health measures. From 2020 to 2021, strategies to mitigate COVID-19 such as masking and distancing effectively reduced the case burden of RSV and HMPV (262). With loosening of these restrictions in the summer of 2021, there was an anomalous increase in pediatric RSV cases in the summer months (262). Collectively, these findings illustrate that public health approaches represent a cost-effective and, in the absence of a pandemic, a possibly underutilized approach towards protecting infants against respiratory viruses.

## Conclusions and Future Directions

Respiratory pathogens, such as RSV, HMPV, and RV, are major contributors to morbidity and mortality in the neonatal population. This may be in part due to the unique immunologic milieu of the neonatal lung, which differs from an adult immune response in practically every cell type. Type 2 immunity predominates in the neonatal lung and, in animal models, contributes to long-term pathology. This is mirrored clinically, as infants exposed to these viruses are at increased risk of development of long-term sequelae such as asthma. However, deeper understanding of the underlying immunologic differences in neonates has the potential to impact how clinicians consider these pathologies, both in the acute and long-term settings. For instance, understanding the pre-existing imbalance of type 1 and type 2 immunity in the neonate could lead to development of immunomodulatory therapeutics to boost the former or suppress the latter. Several pre-clinical models have inhibited type 2 immune factors during the acute immune response and mitigated long-term pathology. Preventing an increased risk of asthma in infants with lower respiratory tract viral infections is a lofty but worthy aspiration. From a basic science perspective, there are many new avenues of neonatal lung biology to explore, such as the roles of the neonatal microbiome (lung and gut) and the use of broad -omics based techniques to elucidate novel aspects of neonatal immunity. While the clinical realm awaits the furthered characterization and promise of translation of these findings, respiratory viral infections will continue to present a significant challenge to neonates and infants. Far too many young children still succumb to these infections, with disproportionate mortality in resource-limited areas of the world, highlighting the need for cost-effective interventions. Although therapeutic options are limited at present, advances in vaccination and monoclonal antibody prophylactics are hoped to translate to increased prevention in neonates.

## Author Contributions

TE - drafted and revised manuscript, generated figures. OP - drafted a section of manuscript, helped generate figures, and revised manuscript. JW - drafting/writing of manuscript. All authors contributed to the article and approved the submitted version.

## Funding

Research reported in this publication was supported by the Eunice Kennedy Shriver National Institute Of Child Health & Human Development of the National Institutes of Health (K12 HD000850, TE), the National Institute of General Medicacal Sciences T32 GM008208 (OP), the National Institute of Allergy and Infectious Diseases (R01 AI085062, JW), and the Henry L. Hillam Foundation (JW).

## Conflict of Interest

The remaining authors declare that the research was conducted in the absence of any commercial or financial relationships that could be construed as a potential conflict of interest.

The handling editor KE declared a shared affiliation with the author OP at the time of review.

## Publisher’s Note

All claims expressed in this article are solely those of the authors and do not necessarily represent those of their affiliated organizations, or those of the publisher, the editors and the reviewers. Any product that may be evaluated in this article, or claim that may be made by its manufacturer, is not guaranteed or endorsed by the publisher.
